# Stress Monitoring of Concrete via Uniaxial Piezoelectric Sensor

**DOI:** 10.3390/s22114041

**Published:** 2022-05-26

**Authors:** Chen Wu, Hong Xiang, Shaofei Jiang, Shenglan Ma

**Affiliations:** 1 College of Civil Engineering, Fuzhou University, Fuzhou 350108, China; xianghong@fjut.edu.cn (H.X.); cejsf@fzu.edu.cn (S.J.); 2 Fujian Provincial Key Laboratory of Advanced Technology and Informatization in Civil Engineering, Fujian University of Technology, Fuzhou 350118, China; mashenglan@fjut.edu.cn

**Keywords:** stress monitoring, concrete stress, uniaxial piezoelectric sensor, work performance, reinforced concrete (RC)

## Abstract

The uniaxial piezoelectric sensor was developed to overcome the problem of reflecting the output charge of the piezoelectric element as a combination of vectors in the three stress directions. The work performance of the uniaxial piezoelectric sensor under varying load patterns and load rates was investigated. The sensor was embedded in concrete to monitor stress, and its elastic modulus was used as the intermediate bridge to establish the correlation between the embedded sensor and the external sensor. Furthermore, a correction factor for the charge transformation strain was suggested to overcome the mismatching of the sensor’s medium and the concrete. Considering related circumstances, a new stress monitoring method based on a uniaxial piezoelectric sensor was proposed, which can achieve stress whole-process monitoring in concrete and confining stress monitoring in the reinforced concrete column. The results reveal that through the proposed method, the output charge curve of the sensor has a substantial overlap with the stress waveform and high fitting linearity. The work performance of the sensor was stable, and its sensitivity was not affected by loading rate and load pattern. The sensor was embedded in concrete and can coordinate with the concrete deformation. The correction factor of strain obtained by the sensor embedded in concrete was 1.07. The relationship between the charge produced by the embedded sensor and its external calibration sensitivity may be used to implement the whole process of stress monitoring in concrete.

## 1. Introduction

Stress monitoring in concrete under various loads has always been one of the essential topics of interest in the construction industry. Reliable stress monitoring methods are significant for concrete confinement mechanisms [1,2,3], mass concrete cracking [4,5], and component bearing capacity [6,7,8,9]. It is commonly acquired indirectly by attaching strain gauges on the concrete surface [10,11]; however, with this approach, it is challenging to comprehend the stress distribution within the concrete structure precisely. Embedded concrete strain gauges [12,13] and fiber Bragg gratings [14,15] are other reliable monitoring methods, yet they are quite costly. Therefore, it is vital to develop new sensors to monitor the stress in concrete in a more effective and less expensive way.

The use of intelligent materials to monitor the performance of civil engineering constructions has become one of the research hotspots in the field of civil engineering in recent years due to the tremendous development of novel sensing technologies. Piezoelectric materials have attracted the attention of researchers to monitor stress in concrete with their advantages, such as sensing and driving functionalities, fast response speed, stable performance, and low price, and various methods have been proposed in this regard.

Some researchers incorporated pressure-sensitive materials into cement mortar to form cement-based pressure-sensitive sensors [16,17,18,19,20,21], which have excellent interface compatibility with concrete and are suitable for monitoring uniaxial and elastic stress states; however, they are not suitable for monitoring multidirectional and elastic–plastic stress states. Song et al. [22] proposed a cement-based piezoelectric smart aggregate (SA) comprised of a lead zirconate titanate (PZT) patch covered by a waterproof substance sandwiched in a cement mortar block in their study. SA was applied to the collision of overheight vehicles on bridges monitoring [22,23] and road traffic load monitoring [24]. Yang [25] embedded SA into concrete specimens, calibrated SA through the uniaxial compressive strength test, and applied it to the stress monitoring of concrete columns under low amplitude high-frequency load. Liu et al. [26] calibrated the external sensitivity of SA under a variety of load conditions, including impact, sine, square wave, and irregularity. The research above shows that the sensor’s output voltage has an excellent linear relationship with stress; however, in the case of cement mortar usage to produce SA, the compressive strength is rather low, and SA cannot withstand high-strength stress, and is easily crushed. Hou et al. [27,28,29] developed granite-based SA with a strength of up to 100 MPa using granite block instead of cement or mortar base to meet high-strength stress monitoring needs. In combination with the developed charge amplifier, Zhang et al. [30,31] accomplished external calibration of this SA within the range of 45 MPa compressive strength. Du et al. [32] embedded this SA into a quartz sand-filled steel tube column to provide inside calibration sensitivity. Using this, the stress in the quartz sand-filled steel tube column was monitored under impact load. Zhang et al. [33,34] established the relationship of concrete stress and SA output voltage via a cylinder compression test, thereby conducting stress process monitoring of SA inside the column during earthquake action.

To summarize, SA which take advantage of the positive piezoelectric effect have been developed by various researchers for internal stress measurement. However, there are often matching errors between the sensor and the embedded medium; therefore, it is essential to establish the correlation between the sensor’s sensitivity inside and outside of calibration. Moreover, when embedded in concrete, the piezoelectric stress sensor exhibits a complicated state of stress, and the existing sensor transfers lateral stress; thus, the accumulated charge and stress cannot linearly correspond. A detailed explanation can be found in Section 2.1. Considering all this, it is vital to develop a piezoelectric stress sensor to isolate the lateral force.

In this paper, the existing sensor was improved depending on the working mechanism of piezoelectric components in the piezoelectric stress sensor, and a piezoelectric stress sensor was proposed to isolate the lateral force. The novel sensor was calibrated internally and externally, and the sensitivity correlation of sensor calibration was established on the inside and outside. In this context, the concrete stress monitoring method was proposed and used throughout monitoring the whole process stress in concrete and monitoring the confining stress in the reinforced concrete (RC) column to verify the reliability of the proposed method.

## 2. Fundamental Principle of Piezoelectric Stress Sensor

### 2.1. Basic Theory of Piezoelectric Materials

The sensing element of the piezoelectric stress sensor is primarily PZT, which possesses positive and negative piezoelectric effects. When the PZT is deformed by an external force applied in the polarization direction, the top and lower surfaces generate opposite polarity charges, namely the “positive piezoelectric effect”. The piezoelectric stress sensor works on the principle of using the positive piezoelectric effect of the PZT to extract stress information from the structure via measuring the output charge of the PZT embedded in the structure.

The piezoelectric effect reflects the connection between elastic and dielectric characteristics of the crystal. In this regard, the interaction coefficient between the system’s mechanical and electrical quantities should be related to the corresponding state. Precisely determining the relationship of each quantity can be achieved with piezoelectric equations established via analyzing the relationship of physical quantities (stress, electric field intensity, strain, and electric displacement vector, etc.) [35].

PZT in the piezoelectric stress sensor does not require an external power source, and the electrical boundary condition is an electrical short circuit, with a specified strain on the upper and lower surfaces required; therefore, the mechanical boundary condition relies on mechanical freedom. The piezoelectric equation that corresponds to this is as follows:

(1) $$D_{i}=e_{ij}^{\sigma }E_{j}+d_{im}^{d}\sigma_{m}$$

where ***D****_i_* is the electric displacement vector (3 × 1), $e_{ij}^{\sigma }$ is the permittivity matrix (3 × 3), $E_{j}$ is the electric field intensity vector (3 × 1), $d_{im}^{d}$ is the piezoelectric coefficients (3 × 6), and $\sigma_{m}$ is the stress vector (6 × 1). In the equation, *i* = 1~3, *j* = 1~3, *m* = 1~6.

The above equation indicates that the change in the three stress parameters will cause the electric displacement of the PZT to change. To simplify the study, the existing literature [32,36,37] set the $\sigma_{1}$ = $\sigma_{2}$ = 0 in order to only consider single-direction sensing; thus, the correlation between electrical displacement and stress can be further simplified into the following linear relationship:

(2) $$D_{3}=d_{33}\sigma_{3}$$

When PZT is employed directly to monitor stress in the medium, the output charge represents the vector combination of stress in three directions. Concrete is confined in three directions in a steel pipe or steel section. Although the present piezoelectric stress sensor can transfer stress in three directions, it cannot distinguish the proportioning of stress into directions, resulting in a linear imbalance between the collected charge and the stress. Therefore, it is vital to design a piezoelectric stress sensor under uniaxial stress to isolate the force in the directions of 1 and 2, while also only receiving axial pressure in order to create a linear connection between stress and electric displacement, as stated in Equation (2).

### 2.2. Measuring Principle of Piezoelectric Stress Sensor

Since PZT is an active capacitor in the piezoelectric stress sensor, it has high internal resistance and low output energy. Therefore, the piezoelectric stress sensor measurement circuit is typically required to be coupled to a high input impedance preamplifier. The first of its functions is to amplify the weak signal produced by the sensor to make it suitable for measurement, while the second is to convert the high output impedance of the piezoelectric sensor to a low output impedance. Since piezoelectric sensors can output voltage signals or charge signals, preamplifiers can be classified as either voltage or charge amplifiers. However, since the length of the cable in the voltage amplifier circuit influences the voltage amplification coefficient and the low-frequency response is not optimal, a charge amplifier is frequently utilized [36]. The charge amplifier used in the current study is Kistler5080, produced by the Swiss Kistler Company.

## 3. Development and External Calibration Performance Analysis of Uniaxial Piezoelectric Sensor

**Figure 1:**
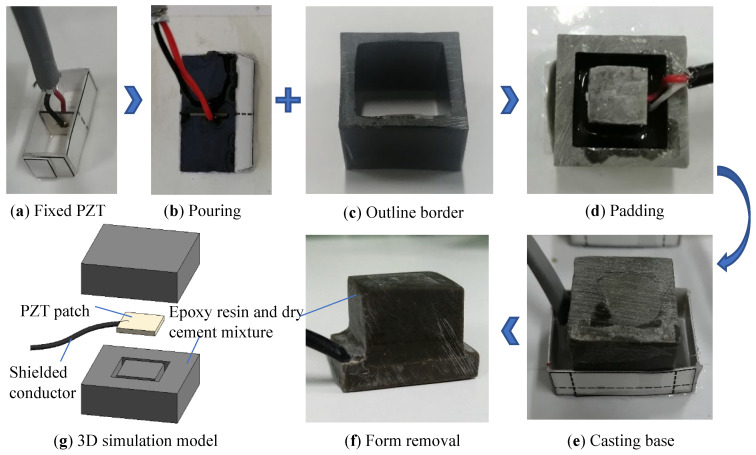
The preparation details of the uniaxial piezoelectric sensor.

To address the issue that the present piezoelectric stress sensor is influenced by lateral confinement, the PZT patch’s perimeter is left blank, and only axial contact and force is applied. The PZT patch of this sensor is solely subjected to axial force and efficiently isolates the lateral force to detect the axial stress inside the concrete; this form of the sensor is termed a uniaxial piezoelectric sensor (Figure 1). The sensor is made by pouring an epoxy resin and dry cement mixture (weight ratio 10:1). The preparation of the developed uniaxial piezoelectric sensor is shown in Figure 1. The PZT patch adopts the PZT-8 model with a minor piezoelectric strain constant. The detailed parameters are shown in Table 1. The sensor size was 20 mm × 20 mm × 20 mm, and the base size was 28 mm × 20 mm × 5 mm.

**Figure 2:**
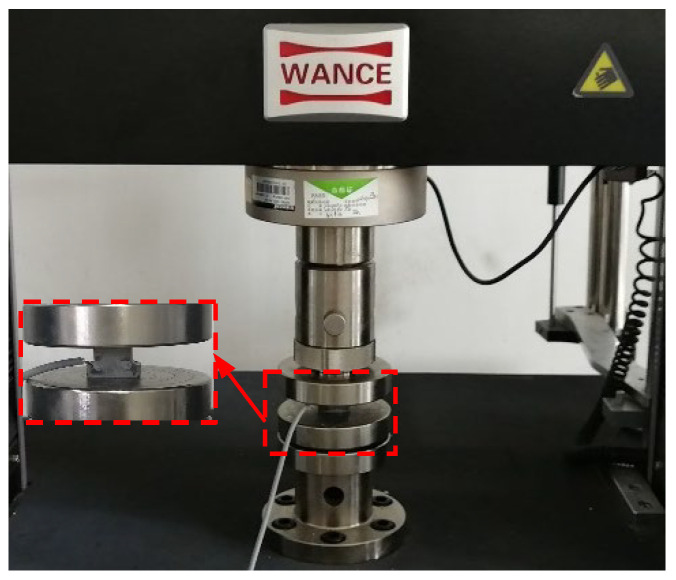
Testing machine for external calibration.

The suggested sensor’s sensitivity was calibrated under four distinct load cases, in which the load patterns included triangular repetition, trapezoid repetition, and stepwise repetition. The impact of loading rate was also considered. Different load cases are presented in Table 2. A stress control scheme was applied. The sensor was calibrated using ETM105D microcomputer control electron universal testing machines (Figure 2).

**Figure 3:**
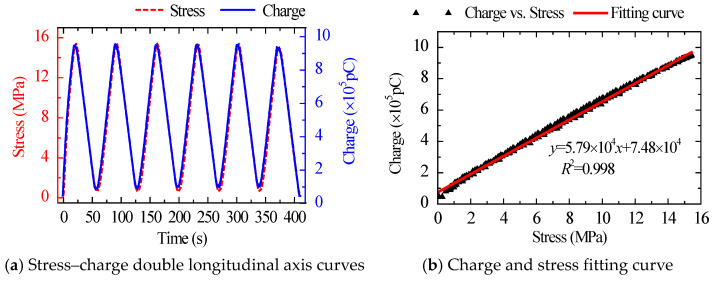
Stress–charge double longitudinal axis curves and fitting curve under Case 1.

**Figure 4:**
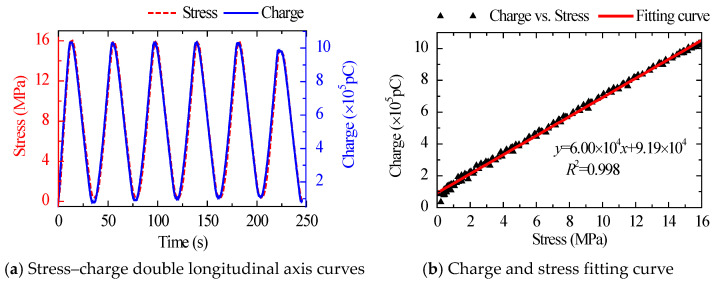
Stress–charge double longitudinal axis curves and fitting curve under Case 2.

The stress–charge dual longitudinal axis curves and fitting curves for the same amplitude and various loading rates are shown in Figure 3 and Figure 4. The charge curve produced by the sensor had a high degree of overlap with the applied stress waveform in both amplitude and phase, and their linear correlation coefficient reached a high level of linearity of 0.998. The results show that the sensor has good response to external load and high linearity between external input stress and output charge.

**Figure 5:**
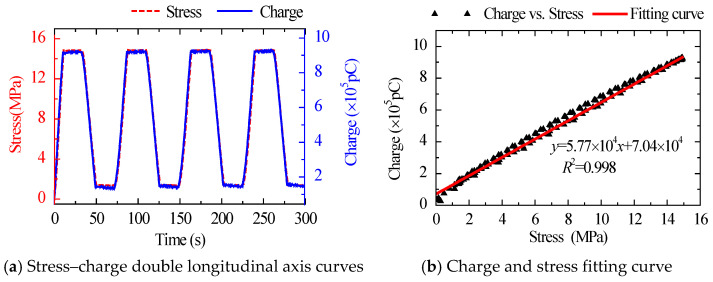
Stress–charge double longitudinal axis curves and fitting curve under Case 3.

**Figure 6:**
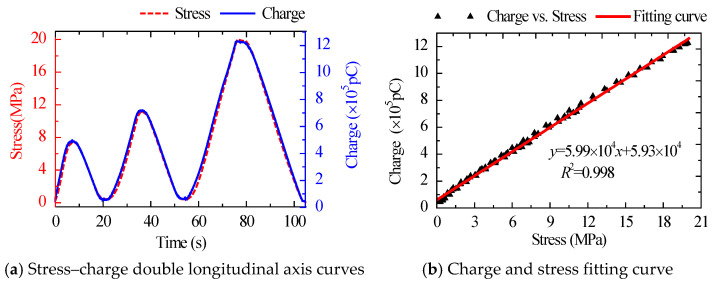
Stress–charge double longitudinal axis curves and fitting curve under Case 4.

The stress–charge dual longitudinal axis curves and fitting curves of various load patterns are shown in Figure 4, Figure 5 and Figure 6. The sensor’s sensitivity error was minimal under different load patterns, with a maximum error of around 4.0%. The findings reveal that the sensitivity of the suggested sensor was little affected by load patterns and rates, and the performance of the sensor was stable.

## 4. Concrete Stress Monitoring Method Based on the Uniaxial Piezoelectric Sensor

**Figure 7:**
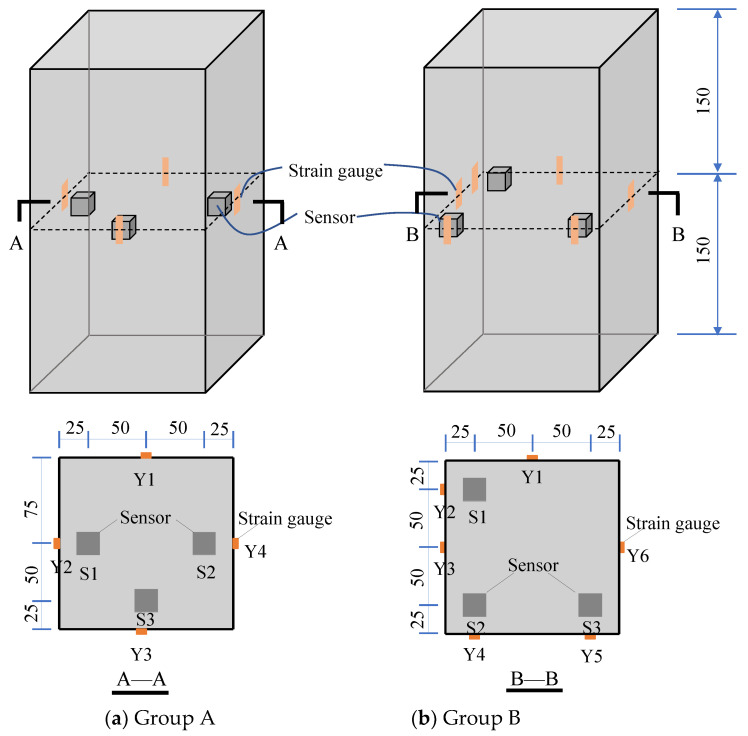
Sensors and strain gauges layout (unit: mm).

To monitor concrete stress under external loads, twelve uniaxial piezoelectric sensors were embedded in the 1/2 height of four different C35 concrete specimens with dimensions of 150 mm × 150 mm × 300 mm (length × width × height). The external calibration sensitivities of twelve sensors were measured according to the triangular loading form in Section 3, and the results are shown in Table 3. Considering the heterogeneity and discreteness of concrete, the sensors were embedded at the corners and edges of the two groups of specimens, respectively, and strain gauges were attached on the corresponding concrete surfaces to establish the layout scheme illustrated in Figure 7. The strain in the sensor’s embedded concrete surface layer was assumed to be equal to that on the corresponding concrete surface, and the strain given by the strain gauge was used as the calibration reference for the embedded sensor.

In order to study the effect of embedded sensors on the mechanical properties of the specimens, the average compressive strengths of the specimens with and without the embedded sensors were compared. The average compressive strength of the embedded sensor specimen was 28.07 MPa, and the average compressive strength of the without-sensor specimen was 29.40 MPa. The errors were as small as 4.5%. Therefore, the influence of embedded sensors on the mechanical properties of the specimen is not considered in this paper.

**Figure 8:**
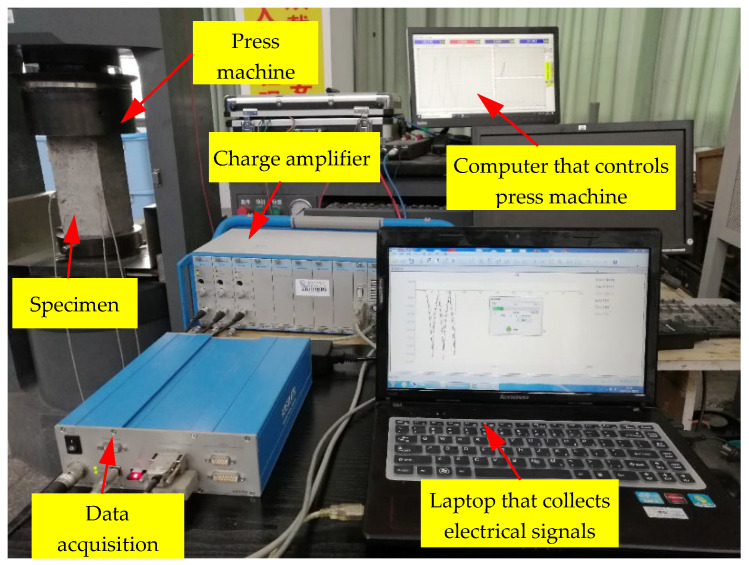
Test devices and data acquisition system.

**Figure 9:**
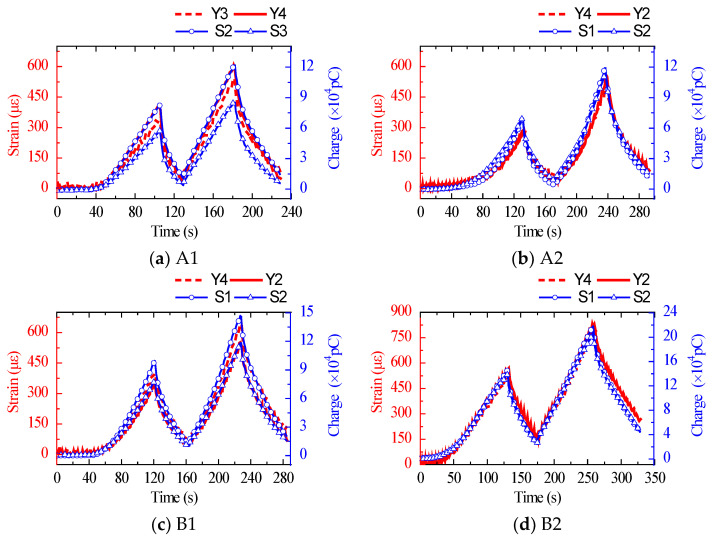
The surface strain of specimens and charge obtained from the embedded sensor in dual longitudinal axes curves.

The test was carried out on a 3000 kN electro-hydraulic servo press machine with a displacement control loading rate of 0.3 mm/min. The maximum average stress was restricted to 15 MPa in order to maintain the concrete within the elastic range. The test devices and data acquisition system are shown in Figure 8. The dual longitudinal axis curve of the strain obtained by the strain gauge attached to the concrete surface and the charge obtained from the embedded sensor are given in Figure 9. The charge curve was consistent with the overall trend of the strain curve without hysteresis. The findings demonstrate that the suggested sensor could coordinate with concrete deformation and monitor changes in concrete internal force.

**Figure 10:**
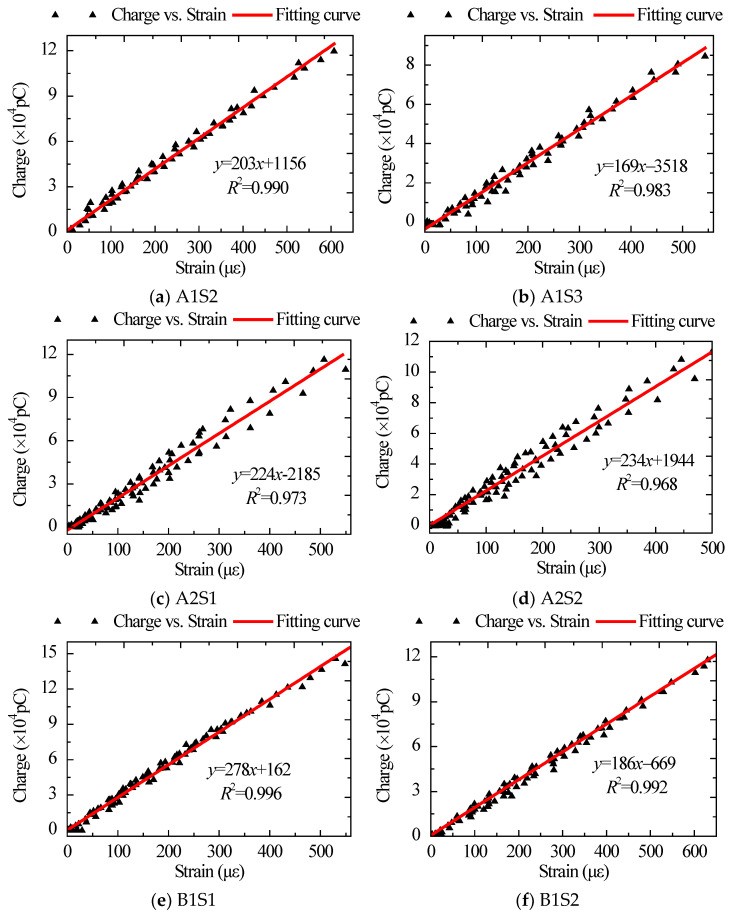
Fitting curves of charge obtained by embedded sensor and concrete surface strain.

From the fitting curve of the charge obtained by the embedded sensor and the concrete surface strain in Figure 10, it can be seen that the charge had an approximately linear relationship with the concrete surface strain.

The embedded sensor’s physical and mechanical properties differ from those of the surrounding concrete, which changes the original stress field of the concrete, causes stress concentration and redistribution, and results in matching errors [38]. For the purpose of eliminating the above “matching error”, the matching coefficient was often used to correct the sensitivity of the sensor. It should be related to the concrete elastic modulus, considering the stress transformation’s direct use. The elastic modulus of concrete ceases to be linear and does not display a constant value when it enters the elastic–plastic behavior stage. Therefore, finding a quantitative link between the matching coefficient and the elastic modulus of concrete is complicated. Therefore, this paper assumes that the sensor is always intact and elastic in the loading process, and that its elastic modulus is a constant value. To establish the correlation between the embedded sensor and the external sensor, the sensor’s elastic modulus was used as an intermediary bridge. The relationship between them was established using the following steps.

Firstly, the sensitivity (*K*_1_) of the sensor’s external calibration was converted into an expression related to strain, as shown in Equation (3). The converted strain ($\varepsilon_{2}^{\prime }$) of embedded concrete of a uniaxial piezoelectric sensor under external load was thereby established, as shown in Equation (4):

(3) $$\alpha_{1}=\frac{Q_{1}}{\varepsilon_{1}}=\frac{Q_{1}E_{\mathrm{ex}}}{\sigma_{1}}=K_{1}E_{\mathrm{ex}}$$

(4) $$\varepsilon_{2}^{\prime }=\frac{Q_{2}}{\alpha_{1}}=\frac{Q_{2}}{K_{1}E_{\mathrm{ex}}}$$

where *Q*_2_ is the ratio of charge to strain obtained by the external sensor, *Q*_1_ is the charge produced by the sensor, *ε*_1_ is the strain applied to the sensor, *σ*_1_ is the stress applied to the sensor, *E*_ex_ is the elastic modulus of the sensor, and *E*_ex_ = 2913 MPa.

Secondly, the embedded sensitivity (*Q*_2_) of the sensor was calculated as the ratio of the charge produced by the embedded concrete of the sensor to the surface strain of the concrete, as given in Equation (5):

(5) $$\alpha_{2}=\frac{Q_{2}}{\varepsilon_{2}}$$

where *Q*_2_ is the charge produced by the embedded concrete of the sensor, and *ε*_2_ is the strain of concrete surface under external load.

Thirdly, the ratio between the converted strain of the embedded sensor and the concrete surface strain was defined as the correction factor (γ), which was absolutely equal to the ratio of *Q*_1_ and *Q*_2_ according to Equation (6). Therefore, *Q*_1_ and *Q*_2_ were used to deduce γ backwards. Table 4 shows *Q*_1_, *α*_2_, and γ of the above twelve sensors. Statistical analysis showed that the mean, standard deviation, and coefficient of variation value of correction factor were 1.07, 0.092, and 0.086, respectively. Therefore, the correction factor of 1.07 was recommended.

(6) $$\gamma =\frac{\varepsilon_{2}^{\prime }}{\varepsilon_{2}}=\frac{Q_{2}}{\alpha_{1}\varepsilon_{2}}=\frac{\alpha_{2}}{\alpha_{1}}$$

Fourthly, Equation (7) demonstrates the connection between the internal concrete strain monitored by the proposed sensor and the external calibration sensitivity.

(7) $$\varepsilon_{2}=\frac{Q_{2}}{\gamma \alpha_{1}}=\frac{Q_{2}}{\gamma K_{1}E_{\mathrm{ex}}}$$

Finally, the internal concrete strain is substituted into the constitutive model of concrete [39] to obtain the internal stress of concrete.

**Figure 11:**
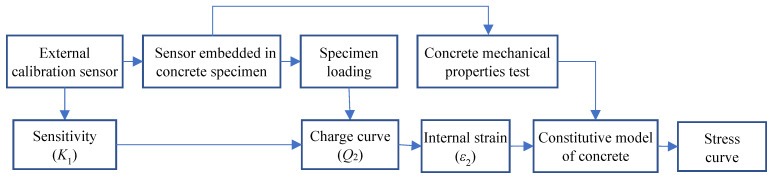
Flow chart of concrete stress monitoring based on the uniaxial piezoelectric sensor.

To summarize, a practical stress monitoring system based on the uniaxial piezoelectric sensor was proposed, and the detailed process is depicted in Figure 11.

## 5. Application and Verification of Monitoring Method

### 5.1. Monitoring the Stress Whole Process in Concrete Specimen

**Figure 12:**
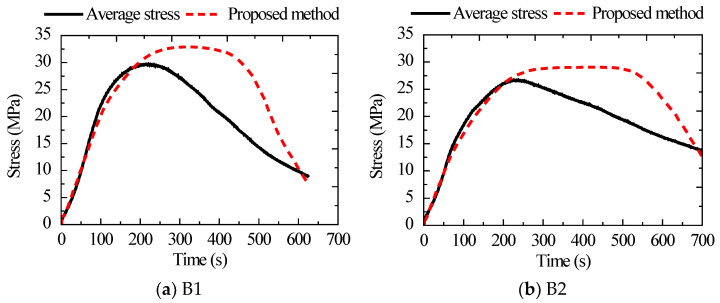
Comparison between the average stress curve and the findings of the suggested method.

The above four specimens were loaded until concrete crushing to obtain the corresponding load curve and charge curve. The charge curve was converted into a stress curve according to the proposed method, and the average value of three sensors was taken as the stress curve of the proposed method. The ratio of the applied load to the cross-sectional area of the specimen was taken as the average stress curve of the specimen. The average stress curve was compared with the findings of the suggested method, as shown in Figure 12, using the data results of Group B as an example. The suggested method’s stress curve was shown to be in better agreement with the specimen’s average stress curve before reaching maximum stress, with average errors of roughly 6.3% and 5.9%, respectively. Observed results reveal the effectiveness of the proposed method for monitoring stress in concrete. Furthermore, the monitored stress after reaching the maximum stress was larger than the average stress and the error between them which increased gradually. This is mainly because the specimen section area was basically intact before reaching the maximum stress. After reaching maximum stress, the surface concrete gradually peeled off and lost its bearing capacity. In addition, the reduction in the cross-sectional area was ignored when calculating the average stress, resulting in minor stress obtained.

### 5.2. Monitoring the RC Column’s Confining Stress

**Figure 13:**
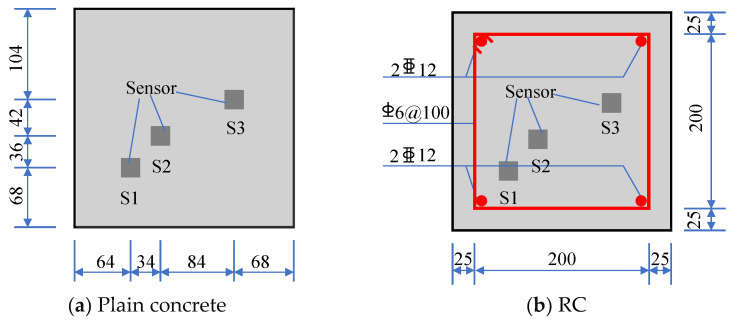
Sensor layout, column section size, and reinforcement (unit: mm).

The proposed sensors were embedded in both RC and plain concrete columns, and the maximum stress of the core concrete of the two columns was compared to observe the confinement effect of the reinforcement on the concrete under axial compression. The column height was 900 mm. The sensor layout, column section size, and reinforcement are shown in Figure 13. 

**Figure 14:**
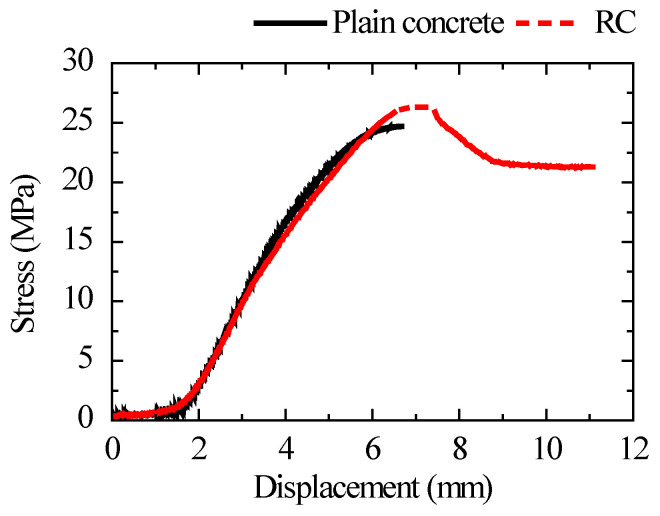
Comparison of stress curves of plain concrete and RC.

According to the mechanical properties test of materials, the yield strengths of longitudinal reinforcement, stirrup, and the axial compressive strength of concrete were 357 MPa, 433 MPa, and 24.91 MPa, respectively. The external calibration sensitivities of six sensors are shown in Table 5. Until the concrete was crushed, the columns were subjected to displacement loading at a rate of 0.3 mm/min. The stress obtained by sensor monitoring, based on the concrete stress monitoring method proposed in Chapter 3, is shown in Figure 14. 

The stress–displacement curve of the RC column was close to that of plain concrete before the displacement was loaded to about 6 mm. The stress–displacement curve of the RC column gradually became greater than that of plain concrete after the displacement was loaded to around 6 mm. The ratio of the maximum stress in RC to plain concrete was 1.064.

According to Mander’s confinement theory [40], the ratio of the compressive strength of confined concrete to that of unconfined concrete represents the concrete strength increase factor. The maximum stress obtained by the sensor embedded in RC was the compressive strength of confined concrete, and the maximum stress obtained by monitoring the sensor embedded in plain concrete was the compressive strength of unconfined concrete, according to the uniaxial force characteristics of the developed sensor. The concrete strength increment factor of reinforced confined concrete was 1.075, according to Mander’s theoretical formula, and the error between monitoring and theory was nearly 1.0%. The dependability of the proposed monitoring method is further detailed.

## 6. Conclusions

A uniaxial piezoelectric sensor only subjected to axial force was proposed based on the PZT working mechanism. The elastic modulus of the sensor was proposed as an intermediate bridge to establish the correlation between the embedded sensor and the external sensor, given that the sensor’s medium did not match the concrete and there was a matching error. Furthermore, the strain obtained by the strain gauge was taken as the embedded sensor’s calibration datum, and a suggested correction factor of strain obtained by the sensor embedded in concrete was provided. On this basis, a concrete stress monitoring method based on the uniaxial piezoelectric sensor was proposed. The suggested method’s dependability was confirmed by monitoring the concrete specimen’s stress during the procedure and the confining stress of the RC column. The following conclusions were obtained:

(1) The proposed sensor had high linearity between applied stress and output charge and responded well to an external load. In addition, the loading rate and load pattern did not affect the sensor’s sensitivity, and its work performance was stable.
(2) The charge curve obtained by the embedded concrete sensor was consistent with the overall trend of the concrete surface strain curve, without hysteresis, and the two were positively proportioned in the concrete’s elastic stage. The strain correction factor obtained by the sensor embedded in concrete was 1.07.
(3) The stress curve derived by the suggested method was in good agreement with the average stress curve of the concrete specimen before it reached maximum stress, and the average error between them was less than 6.3%. The error between the concrete strength increase factor of the proposed technique and Mander’s confinement theory was about 1.0%, which verifies the reliability of the proposed method. Future work will investigate the confinement mechanism of steel-confined concrete based on the proposed method.

## Figures and Tables

**Table 1:** PZT-8 parameters.

Performance Category	Property Values	Performance Category	Property Values	Performance Category	Property Values
Size (mm)	8 × 8 × 0.5	Elastic constants Y_11_^E^ (N/m^2^)	1.2 × 10^10^	Piezoelectric strain constant *d*_33_ (C/N)	232 × 10^−12^
Density (kg/m^3^)	7600	Elastic constants Y_33_^E^ (N/m^2^)	7.0 × 10^10^	Piezoelectric strain constant *d*_31_ (C/N)	−90 × 10^−12^
Relative permittivity *ε*_r_	1400	Elastic constants Y_55_^E^ (N/m^2^)	29.4 × 10^10^	Piezoelectric strain constant *d*_15_ (C/N)	378 × 10^−12^

**Table 2:** Different loading cases.

Loading Cases	Load Patterns	Stress Amplitude Range (MPa)	Loading Rate (MPa/s)
Case 1	Triangular repetition	0~16	0.5
Case 2	Triangular repetition	0~16	0.8
Case 3	Trapezoid repetition	0~16	0.8
Case 4	Stepwise repetition	0~20	0.8

**Table 3:** External calibration sensitivities of twelve sensors (*K*_1_).

Group	Specimen Number	Sensor Number	Sensitivity (pC/MPa)	Group	Specimen Number	Sensor Number	Sensitivity (pC/MPa)
Group A	A1	S1	62,574	Group B	B1	S1	77,160
		S2	66,082			S2	66,304
		S3	54,497			S3	62,275
	A2	S1	74,314		B2	S1	72,842
		S2	87,604			S2	71,858
		S3	57,015			S3	82,699

**Table 4:** Correction factor (γ ).

Sensor Number	$\alpha_{1}$ (pC/με)	$\alpha_{2}$ (pC/με)	γ	Sensor Number	$\alpha_{1}$ (pC/με)	$\alpha_{2}$ (pC/με)	γ
A1S1	182	202	1.11	B1S1	216	278	1.24
A1S2	192	203	1.05	B1S2	255	186	0.96
A1S3	159	169	1.06	B1S3	166	181	1.09
A2S1	216	224	1.04	B2S1	212	256	1.21
A2S2	255	234	0.92	B2S2	209	246	1.18
A2S3	166	174	1.05	B2S3	241	263	1.09
				Average value of correction factor		1.07	
				Standard deviation of correction factor		0.092	
				Variation coefficient of correction factor		0.086	

**Table 5:** External calibration sensitivity of six sensors (*K*_1_).

Specimen	Sensor Number	Sensitivity (pC/MPa)	Specimen	Sensor Number	Sensitivity (pC/MPa)
Plain concrete	S1	58,153	RC	S1	59,988
	S2	36,139		S2	38,324
	S3	47,228		S3	47,351

## Data Availability

The data presented in this study are available on request from the corresponding author.
